# Improved virtual extensometer measurement method in complex multi-fracture situation

**DOI:** 10.1038/s41598-022-08393-9

**Published:** 2022-04-26

**Authors:** Jing Chai, Yibo Ouyang, Jinxuan Liu, Dingding Zhang, Wengang Du, Jianfeng Yang, Yongliang Liu, Zhe Ma

**Affiliations:** 1 School of Energy Science and Engineering, Xi’an University of Science and Technology, Xi’an, 710054 Shaanxi China; 2 Key Laboratory of Western Mine Exploitation and Hazard Prevention, Ministry of Education, Xi’an, 710054 Shaanxi China

**Keywords:** Civil engineering, Petrology

## Abstract

To overcome the limitation of the virtual extensometer method in measuring the crack opening displacement (COD) in the process of complex multi-crack propagation of rock, the measurement error of Digital Image Correlation (DIC) local deformation is theoretically analyzed. An improved virtual extensometer method for measuring the COD is proposed, which considers the temporal and spatial characteristics of crack development in the process of complex crack propagation. The accuracy of the proposed method is verified by the strain localization band numerical simulation test and indoor single crack simulation test. Furthermore, the method is applied to the two-dimensional similarity simulation test of simulating complex multi-fractures in rock stratum. The COD obtained by the traditional and improved methods is compared with the measured COD. The results show that in the case of multiple complex cracks, to obtain the COD accurately, the relative distance between the virtual extensometer measuring point and the crack should be greater than half of the sum of the width of the crack strain localization zone and the subset size. With the development of the crack, the relative distance between the virtual extensometer measuring point and the crack should increase with the increase of the width of the crack strain localization zone. The error of the COD measured by the traditional method increases with fracture development, and the maximum is 21.20%. The maximum relative error between the COD measured by the improved method and the measured crack opening is 3.61%. The research results improve the accuracy of the virtual extensometer in measuring the COD under complex multi-crack conditions.

## Introduction

The occurrence and development law and quantitative description of a fracture network in mining rock mass are mine rock mechanics' fundamental and challenging problems^1^. Crack detection based on Digital Image Correlation (DIC) technology is widely used in architecture, structure, material, aerospace, and other fields. The acquisition of fracture parameters such as fracture location, opening, and development length is the premise to study the tested structure's mechanical properties and failure characteristics and plays an intuitive and essential role in the stability evaluation and disaster prediction of geotechnical engineering^2–5^.

DIC is a non-contact measurement method based on the image. The whole field detection of structural deformation is realized by taking photos of the speckle field on the surface of the measured structure and then processing the image. In recent years, DIC is widely used in studying the fracture process of materials with defects such as prefabricated cracks^6–9^. The traditional measurement method of the crack opening displacement obtained by DIC was first proposed by M. A. Sutton et al. in the 1990s^4^. This method is generally called the "virtual extensometer method". The virtual extensometer method is to arrange measuring points symmetrically at a certain distance on both sides of the crack or select a specific size calculation area. Then, the vertical displacement is decomposed along the vertical crack direction, and the difference is calculated as the crack opening displacement. Since then, many scholars at home and abroad have applied this method to the study of fracture propagation to get the fracture mechanics parameters of the research object^10–15^. Yuan Y. et al. sets two measuring points symmetrically on both sides of the potential crack propagation path^16^. Xie H et al. The length of the extensometer should be greater than the subset size plus twice the maximum the COD (crack opening displacement) value^17^. Vormwald M. et al. proposed an experimental technology to measure the crack opening and closing degree in the constant strain amplitude test, place the virtual strain gauge as close to the fatigue crack as possible^18^. S. Rabbolini. et al. moved the virtual strain gauge between the cyclic plastic zone and the notch in order to obtain more accurate results^19^. Patriarca L. et al. proposed that the nominal length of the extensometer shall be as close as possible to zero correspondings to the crack surface. Since this is an ideal situation, considering the problems related to the selected subset and crack area, the end of the extensometer should be as close to the crack surface as possible^20^. The relative position of the measuring point and crack is directly related to the accuracy of DIC measurement results and then affects the judgment of material properties^21^. The different analyses of measuring points to varying distances from fractures in calculating the crack opening displacement have not been carried out. Although scholars have made many valuable achievements in rock fracture research by using DIC, there are still disputes about the layout position of measuring points when using the virtual extensometer method to measure the crack opening displacement.

For some simple single fracture activities, the traditional virtual extensometer method has significant advantages. Still, for the complex fracture propagation process in rock, there are some limitations: The width of the strain localization zone is often much larger than the fracture width, and the traditional virtual extensometer method is challenging to obtain the accurate position of fracture only through the localization zone position. For heterogeneous materials, the strain localization band is not always symmetrically distributed around the crack. Using the traditional virtual extensometer method, and the measuring points on one side of the crack may be located in the non-uniform deformation region of the strain localization zone, increasing the measurement error. With the development of fractures, the width of the local strain band is constantly changing. The traditional virtual extensometer method ignores the temporal and spatial characteristics of fracture development in heterogeneous materials and fails to reasonably adjust the distance between the measuring point and the fracture according to the actual result of the fracture.

Given this, through the theoretical analysis of the measurement accuracy of DIC local deformation, an improved DIC crack opening measurement method is proposed. The accuracy of this method is verified by strain localization band numerical simulation test and indoor single crack simulation test. The method is applied to the two-dimensional similarity simulation test of simulating complex multi fractures in rock stratum, and the influence of different measurement points on the measurement of crack opening by DIC is discussed. The crack opening obtained by the virtual extensometer method and the improved method is compared with the measured crack opening, and the reliability of the improved method is verified. The results provide an experimental and theoretical basis for further studying the fracture characteristics of rock materials by DIC.

## Principle of measurement method

### Principle of DIC strain measurement

**Figure 1:**
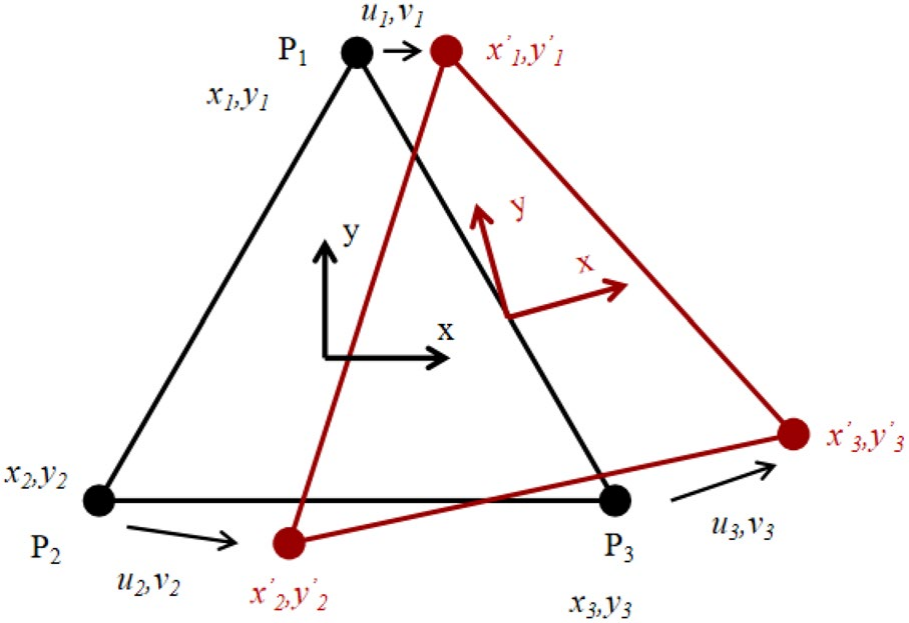
Calculation principle of digital image correlation strain.

Digital Image Correlation (DIC) technique is an optical method based on non-contact images, which uses Digital images to obtain the full-field displacement and strain responses of structures. It has been applied in rock mechanics tests by many scholars. DIC technology uses an industrial camera to obtain speckle images on the surface of an object^22–26^. It then carries out image analysis based on the algorithm to quantitatively extract deformation information such as three-dimensional coordinates, displacement field, and strain field on the surface of the measured structure. DIC obtains the displacement of measurement point *P*_*i*_ (*i* = 1,2,3) by calculating the displacement difference between the center points of the subset before and after the deformed image, as shown in Fig. 1.

DIC strain calculation is essentially the derivative of displacement, and the variation of subset measurement points in space can be expressed by the gradient matrix ***F***:

3 $$ \left[ {\begin{array}{*{20}c} {x_{i}^{^{\prime}} } \\ {y_{i}^{^{\prime}} } \\ \end{array} } \right] = \left[ {\begin{array}{*{20}c} {u_{i} } \\ {v_{i} } \\ \end{array} } \right] + \left[ {\begin{array}{*{20}c} {F_{11} } & {F_{12} } \\ {F_{21} } & {F_{22} } \\ \end{array} } \right] \cdot \left[ {\begin{array}{*{20}c} {x_{i} } \\ {y_{i} } \\ \end{array} } \right] $$

There are six unknowns: (*u*_*i*_*, v*_*i*_*, F*_*11*_*, F*_*12*_*, F*_*21*_*, F*_*22*_). Therefore, deformation information of at least three measuring points is needed. The least-square method is used to calculate the deformation tensor ***U***^35^:

4 $$ U = \sqrt {F^{T} F} = \left[ {\begin{array}{*{20}c} {\varepsilon_{{\text{x}}} + 1} & {\varepsilon_{{{\text{xy}}}} } \\ {\varepsilon_{yx} } & {\varepsilon_{{\text{y}}} { + }1} \\ \end{array} } \right] $$

From Eq. (4), the strain of the measured object in x and y directions can be obtained.

### DIC local deformation measurement accuracy

The measurement accuracy of DIC in the local deformation has an important influence on quantifying the strain localization zone, the deformation near the crack, and the determination of the fracture parameters of the crack^27–30^. The calculation parameters affect the displacement measurement results because the shape function does not match the actual deformation. The size and spacing of the subset will have a particular effect on the displacement measurement accuracy and the measurement results of the strain localization band. For example, the width of the strain localization band tends to increase with the increase of subset size or spacing. There have been many effective evaluation methods for the accuracy of DIC measurement of the uniform displacement field. Still, there is a lack of effective evaluation methods and theoretical calculation models for the accuracy of DIC measurement of local deformation. Therefore, it is necessary to analyze the measurement accuracy of local non-uniform deformation measured by DIC.

**Figure 2:**
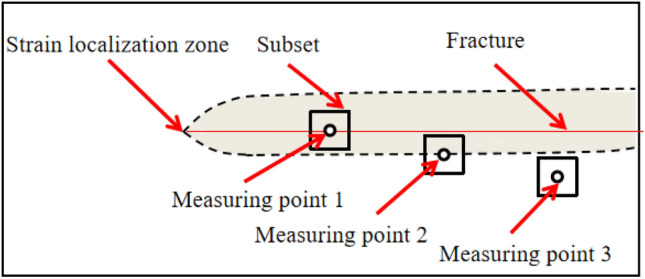
Diagram of deformation around fracture measured by DIC.

According to Sect. 2.1, the essence of the DIC displacement measurement is based on the assumption of continuous deformation of the measured object. When the size of the subset contains four or more speckles and the subset spacing is 1/3 or more of the side length of the subset, the displacement test data of the measurement points located in the uniform deformation area are close to the actual displacement value. Under the same calculation parameters, the displacement test data of the measurement points in the local non-uniform deformation area near the crack are far from the actual displacement. Figure 2 shows a schematic diagram of the deformation around the crack measured by DIC. Measuring point 1 is located on the crack, and its square subset includes the crack area and the local non-uniform deformation area in the strain localization zone. Therefore, the displacement calculated by DIC is not equal to the actual displacement of the crack, and the displacement value of measuring point 1 must be very different from the actual displacement value. The measuring point 2 is arranged along the boundary of the displacement localization zone and covers the displacement localization zone area of non-uniform deformation and the displacement localization zone area of uniform deformation. Therefore, DIC cannot accurately obtain the displacement values of the non-uniform deformation zone and the uniform deformation zone simultaneously. The measured displacement value of measuring point 2 is significantly different from the actual displacement value. The measuring point 3 is located outside the strain localization zone, and the subset only contains the uniform deformation outside the strain localization zone. Therefore, the displacement value of measuring point 3 is consistent with the actual displacement value. The above analysis is the test result when the DIC calculation parameter is that the subset size contains four or more speckles, and the subset spacing is 1 / 3 or more of the subset side length. Of course, this is also the calculation parameter selection principle that must be followed in the accurate measurement of the crack opening displacement by the virtual extensometer method.

## Improved virtual extensometer method

### Improvement method implementation procedure

**Figure 3:**
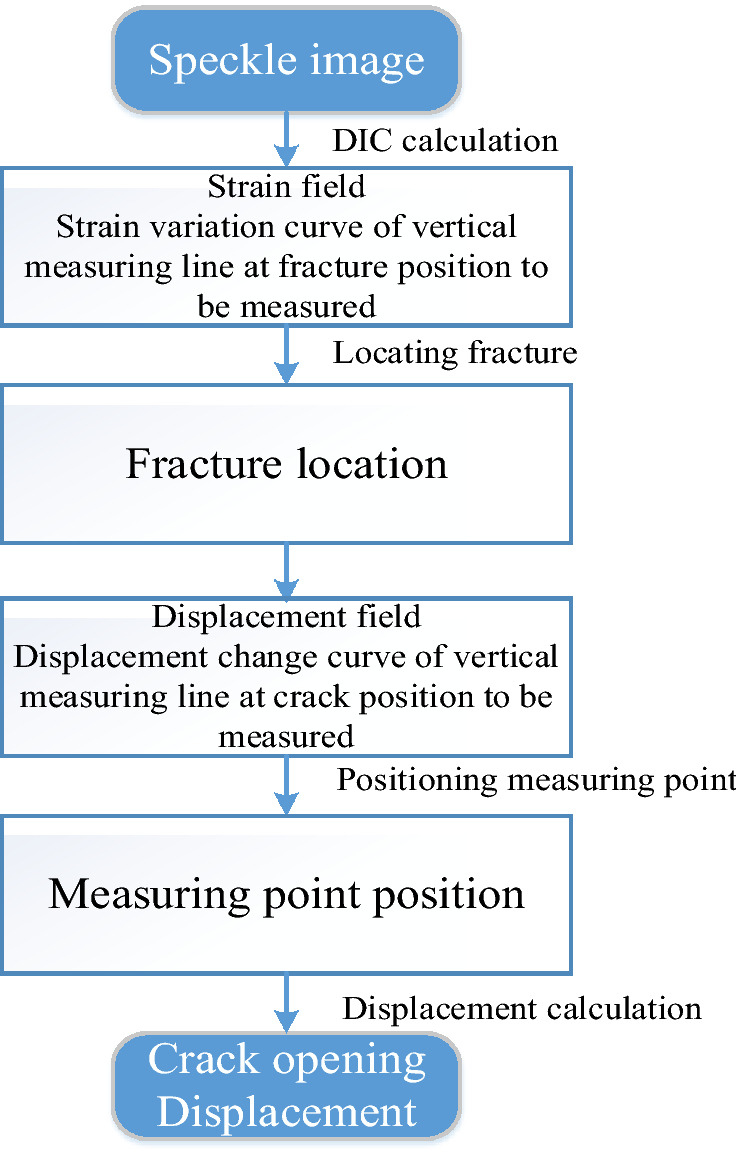
Implementation plan of improved method.

According to the above theoretical analysis of DIC local deformation measurement error, an improved virtual extensometer crack opening measurement method is proposed in this paper. The improved method is based on the strain field measured by DIC, and its scheme is shown in Fig. 3. Firstly, the speckle image is imported, and DIC calculates the strain field cloud map to determine the strain localization zone, that is, the approximate range of cracks. Then the crack position is determined according to the strain change curve of the vertical measurement line at the fracture position to be measured. Then, according to the displacement change curve of the vertical measuring line, the layout position of the measuring points is determined. Finally, the difference between the displacement values of the two measuring points is the crack opening. In the case of multiple complex cracks, to obtain the COD accurately, the relative distance between the virtual extensometer and the crack should be greater than half of the sum of the width of the crack strain localization zone and the subset size. With the development of the crack, the relative distance between the virtual extensometer and the crack should increase with the increase of the width of the crack strain localization zone.

**Figure 4:**
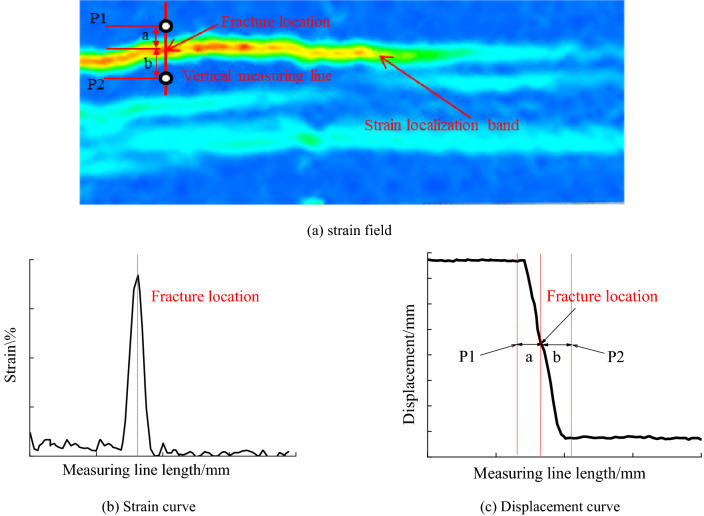
Schematics of improved method.

Figure 4 is a schematic diagram of an improved method. As shown in Fig. 4a, a measuring line perpendicular to the strain localization zone is arranged in the strain field program to locate the crack, and the measuring line is 200 mm long. Figure 4b is a strain change curve at each point along the measurement line measured by DIC. The peak position of the strain curve corresponds to the crack position. Figure 4c shows the displacement change curve of each point along the measuring line measured by DIC, and the displacement curve is distributed in steps as a whole. The area from the origin of the abscissa to the first red dotted line becomes area A. The second red dotted line corresponds to the crack position, the area between the first red dotted line and the third red dotted line becomes area B, and the area from the third red dotted line to the end of the abscissa is area C. Therein, the overall change of displacement in Zone C and Zone A is small, and the measurement line in this zone is located in the uniform deformation zone outside the strain localization band. The displacement in zone B changes sharply, and the measurement line in this zone is located in the local non-uniform deformation zone of the strain localization zone. According to the analysis in Sect. 2 of this paper, the measuring points should be arranged in the uniform deformation area outside the strain localization zone. Measuring points P1 and P2 shall be arranged at any position in areas A and C, respectively. Since the strain localization band is not simply symmetrically distributed about the crack, generally, the distance a between the measurement point P1 and the crack is not equal to the distance b between the measurement point P2 and the crack.

Adjust the direction of the coordinate system so that the Y-axis of the coordinate system is perpendicular to the fracture direction, and calculate the difference between the vertical displacement of measuring points P1 and P2, that is, the crack opening at this point. It should be noted that with the development of cracks, the width of the strain localization zone may change. It is necessary to repeat the above steps to determine the position of measurement points again to ensure measurement accuracy.

For complex and multiple cracks, the layout of measurement points needs to comprehensively consider the influence of other strain localization zones and try to avoid the situation that the measurement points are arranged on the strain localization zone. As shown in Fig. 4a, the measuring point P1 is located outside the strain localization band, and the measuring point P2 is located between the two strain localization bands. In the case of complex multi fractures, in order to accurately obtain the the crack opening displacement, the relative distance between the measuring point of the virtual extensometer and the fracture should be greater than half of the width of the strain localization zone with the change of the fracture. Only in this way can the accuracy of displacement measurement be guaranteed. Compared with the traditional method, the improved method not only considers the influence of strain localization zone and fracture on the displacement error of measuring points but also considers the temporal and spatial characteristics of fracture development in the process of complex fracture propagation, which can realize the reasonable layout of measuring points under multiple cracks and multiple strain localization zones.

The essence of DIC is to obtain the displacement and influence by analyzing the speckle information of the measured object. Therefore, if the measured object can provide a good speckle pattern, the method described in this paper is applicable. Therefore, this method applies to rock mechanics tests, 3D-DIC scenes, and even Volumetric Digital Image Correlation(VDIC). The DIC method is based on continuous deformation testing technology. Therefore, if the speckle on the surface of the measured structure falls off completely, the virtual extensometer method will not be applicable. The falling off of rock mass on the model surface will lead to the loss of speckle on the model surface so that DIC cannot be measured at the lack of speckle on the model. When the number of adjacent cracks or defects in a small area increases, measurement accuracy can be guaranteed as long as the measuring point of the virtual extensometer is not located in the strain localization area and crack area.

### Numerical simulation verification

The displacement or strain of the strain localization band cannot be controlled by physical tests, which will make it difficult to evaluate the measurement accuracy of DIC measurement results^31,32^. Therefore, the simulation diagram in the DIC Challenge (P200_K50_n2 and P200_K100_n2), the number simulation diagram of 2000 × 1000 pixels, was used for analysis with DIC, and the displacement measurement result of a transversal line was obtained and compared with its actual value.

**Figure 5:**
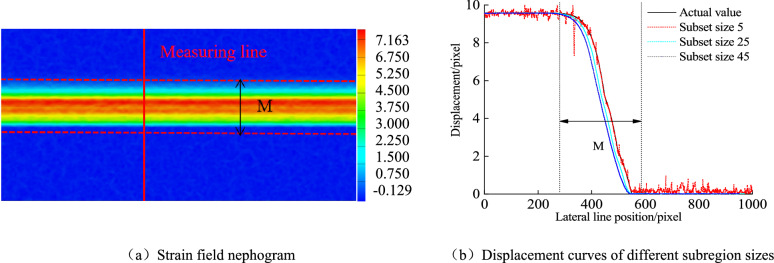
Numerical simulation of strain localization band.

Figure 5b presents the displacement measurement results when the subset size is between 5 pixel × 5 pixel-45 pixel × 45 pixel, and the subset spacing is 1 pixel. When the subset size is between 15 pixel × 15 pixel and 45 pixel × 45 pixel, the deviation between the measured results in the localized band and the actual value gradually increases. Still, the measured results out of the localized band are almost the same as the actual value, which is only affected by the random error. When the subset size is 5 pixel × 5 pixel, the real deformation in the band matches the shape function. Still, for the relatively uniform deformation out of the band, the shape function has appeared matching phenomenon, resulting in large fluctuation of the out-of-band data. With the increase of subset, the undermatching degree of shape function becomes more and more serious. As the increase of the subset will reduce the random error of the displacement measurement results, and the shape function matches the out-of-band deformation, the out-of-band displacement data is close to the real data. At the shear band junction (the measuring line position at 245 mm and 542 mm), DIC measurements do not match the true values well because the subzone covers two different types of in-band and out-band deformation at the strain-localized band junction. For this reason, the localized bandwidth measured by DIC is larger than the true value.

In Fig. 5a, M refers to a distance of 300 pixels between two survey lines more significant than the strain localization influence band range measured by DIC (its width is 293 pixels). In other words, the DIC measured value at the survey line is no longer affected by the strain localization band. When DIC is used to measure the crack opening, as long as the size of the subset is large enough, the accuracy of localized out-of-band displacement measurement can always be guaranteed. The crack opening can be accurately obtained by placing the measuring points or lines in the out-of-band area. On the contrary, if the measuring points or measuring lines are arranged in the zone or at the junction, there will be a significant error in the crack opening. An important conclusion can be found from the results in Fig. 5b. That is, the accuracy of the measured values in the strain localization region can be improved only by reducing the size and spacing of the subsets. In other words, the measurement accuracy of virtual extensometer points in the strain localization region increases with the decrease of subset size and spacing. Numerical simulation experiments verify the correctness of the theoretical analysis and the accuracy of the improved method.

### Laboratory simulation test of single crack

**Figure 6:**
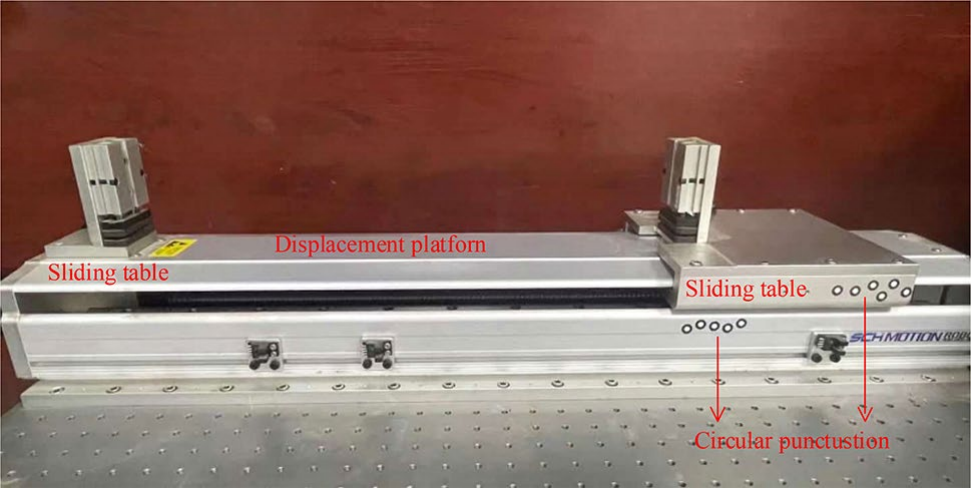
High precision displacement platform.

As shown in Fig. 6, firstly, the speckle model with excellent speckle quality is generated by using the independently developed speckle production software, then printed on two sheets of paper and pasted on the left and right sliding platforms of the high-precision displacement platform, respectively. The translation of the sliding platform is used to simulate the crack opening process. The DIC test system mainly includes an industrial camera (2560 × 1920 pixel), LED light source, computer, and 2D-DIC analysis software. Because the test object is the movement and deformation of two-dimensional plane paper and does not pay attention to the out-of-plane displacement, the selection of 2D-DIC test system in this paper can reduce the test cost and improve the test efficiency. The test platform moves with a displacement gradient of 0.2 mm each time nine times, a total of 1.8 mm. Collect five pictures each time. The magnification is 7.44 pixel/mm. The length of the subset is 66 pixels, and the subset spacing is 22 pixels.

**Figure 7:**
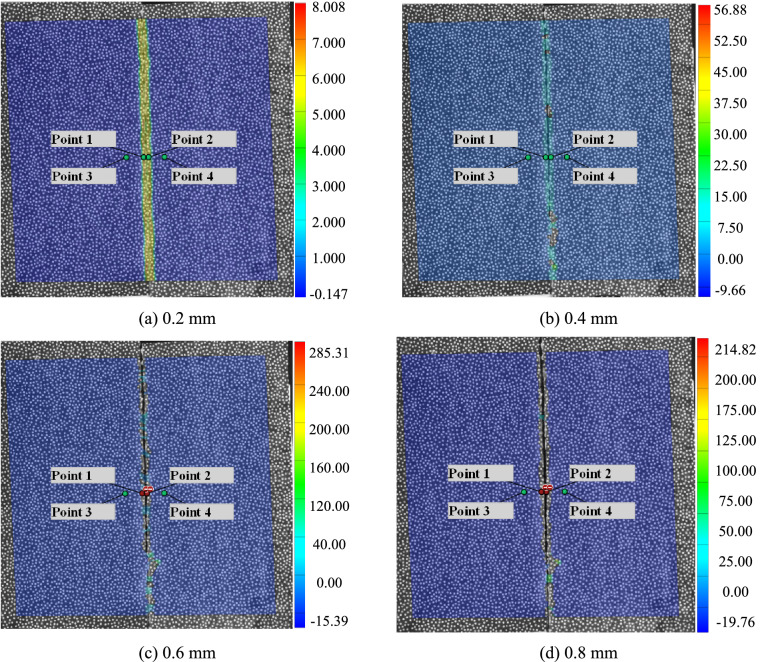
DIC strain nephogram and measuring point layout.

As shown in Fig. 7a, when the crack opening is 0.2 mm, a strain localization band appears centered on the crack, the width of strain localization band is 3.88 mm. As shown in Fig. 7b, when the crack opening is 0.4 mm, a strain localization band appears centered on the crack, the width of strain localization band is 4.01 mm. The measuring points 1 and 2 are arranged in the strain localization zone, and the measuring points 3 and 4 are arranged outside the strain localization zone. Because a single crack is simulated, the change around the crack is uniform deformation, and the measuring point 3 and measuring point 4 are far away from the strain localization zone, the position of the measuring point is not adjusted with the crack opening in the follow-up. With the increase of crack opening, the strain field diagram breaks, so that measuring point 1 and measuring point 2 lose their calculation ability, as shown in Fig. 7c,d.

**Figure 8:**
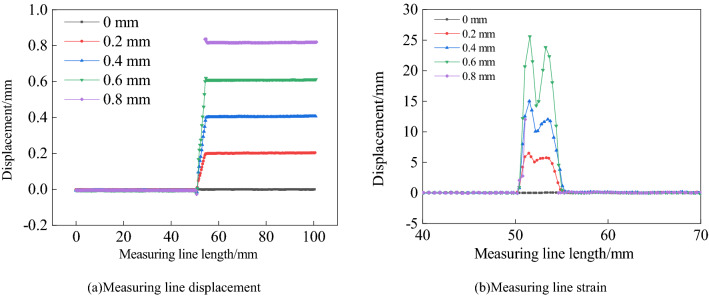
DIC measurement line test results.

A measuring line is arranged in parallel with the position of the measuring point, as shown in Fig. 8a, which is the change of displacement on the length of the measuring line. When the crack opening is 0 mm, there is no displacement on the whole measuring line. When the crack opening is 0.2 mm, it appears as the center, and a step displacement change curve appears, and the step height is the crack opening. Then, the step height increases with the increase of crack opening. Until the crack opening is 0.8 mm, there is no displacement data at the measuring point at the crack position. As shown in Fig. 8b shows the change of strain on the length of the measuring line. With the increase of crack opening, the width of the strain localization band increases gradually. Until the crack opening is 0.8 mm, there is no strain data at the measuring point at the crack position. The strain curve has double convex peaks and the corresponding crack position between the two convex peaks. The length of the strain localization band determined according to the distribution range of the strain curve is 3.62 mm, which is basically consistent with the length of the strain localization band shown in Fig. 7a. With the increase of crack opening displacement, the strain localization width increases.

**Figure 9:**
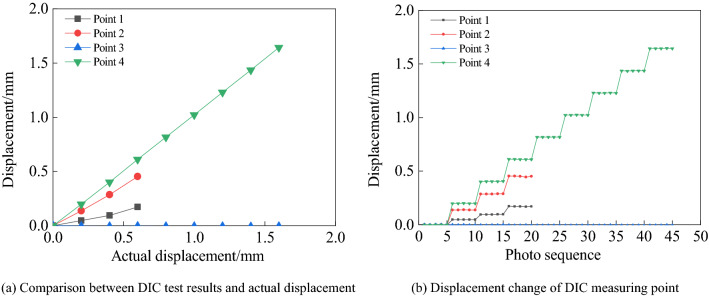
DIC measurement results.

As shown in Fig. 9, the displacement change curves of measuring points 1 ~ 4 are shown respectively, and the abscissa is the photo sequence. Five photos are taken each time, 45 photos in total. It can be seen from the Fig. 9a that the displacement change of measuring point 3 is always 0 mm, which is consistent with reality. This is because the sliding platform of measuring point 1 and measuring point 3 does not move. In contrast, due to the influence of the strain localization band, the displacement of measurement point 1 changes nonlinearly, up to 0.21 mm. The displacement change of measuring point 4 is consistent with the actual displacement, proving the accuracy of DIC measurement. At the same time, the displacement change of measuring point 2 also presents a nonlinear transformation, and the maximum error with the actual displacement is 37%. As shown in Fig. 9b, the displacement of the DIC measuring point rises in steps as a whole.

The test results verify the correctness of the above theoretical analysis. The relative position between the measuring point and the crack directly affects the accuracy of DIC in measuring the crack opening. In other words, the width of strain localization directly affects the layout of measuring points. Of course, the test results verify the improved method's accuracy to measure the crack opening.

## Similarity physical model test

### Model overview

The physical similarity model test reduces engineering problems to the scale of the laboratory model according to a certain similarity ratio. Scientific and reasonable selection of similarity ratio is an essential guarantee for the authenticity and reliability of test results^33–36^. It is required that the size of each part of the experimental model and the prototype shall be reduced or enlarged according to the same scale, that is: "*l*_*p*_* / l*_*m*_ = *C*_*l*_", where *l*_*p*_ is the prototype size, *l*_*m*_ is the model size, and *C*_*l*_ is the geometric similarity constant. According to the selected geological data and the size of the test model frame, the geometric similarity ratio is determined as: "*C*_*l*_ = 1:200", and the geometric size of the model is 1200 × 1200 × 200 mm, the boundary condition is a plane stress model. The bulk density similarity constant is the ratio of the density of prototype material to that of model material. Namely: *C*_*γ*_ = γ_p_ / γ_m_ = 1.56, the stress similarity constant is the stress ratio between prototype and model. Both prototype and model should meet the equilibrium differential equation within the elastic range. The similarity index satisfying the equation is *C*_*σ*_ = *C*_*l*_*C*_*γ*_。The stress similarity constant is obtained by substituting the geometric similarity constant and the bulk density similarity constant *C*_*σ*_ = 312. The stress value obtained from the model test must be transformed into the engineering stress value through the stress similarity consistent. The time similarity consistent is 14.14. In the process of model excavation, the actual mining time of each cycle of the working face is transformed into the model excavation time according to the time similarity constant. After each excavation, it is allowed to stay for 20 min. The following mining cycle can be carried out after the overlying strata and collapsed gangue is fully stable.

**Figure 10:**
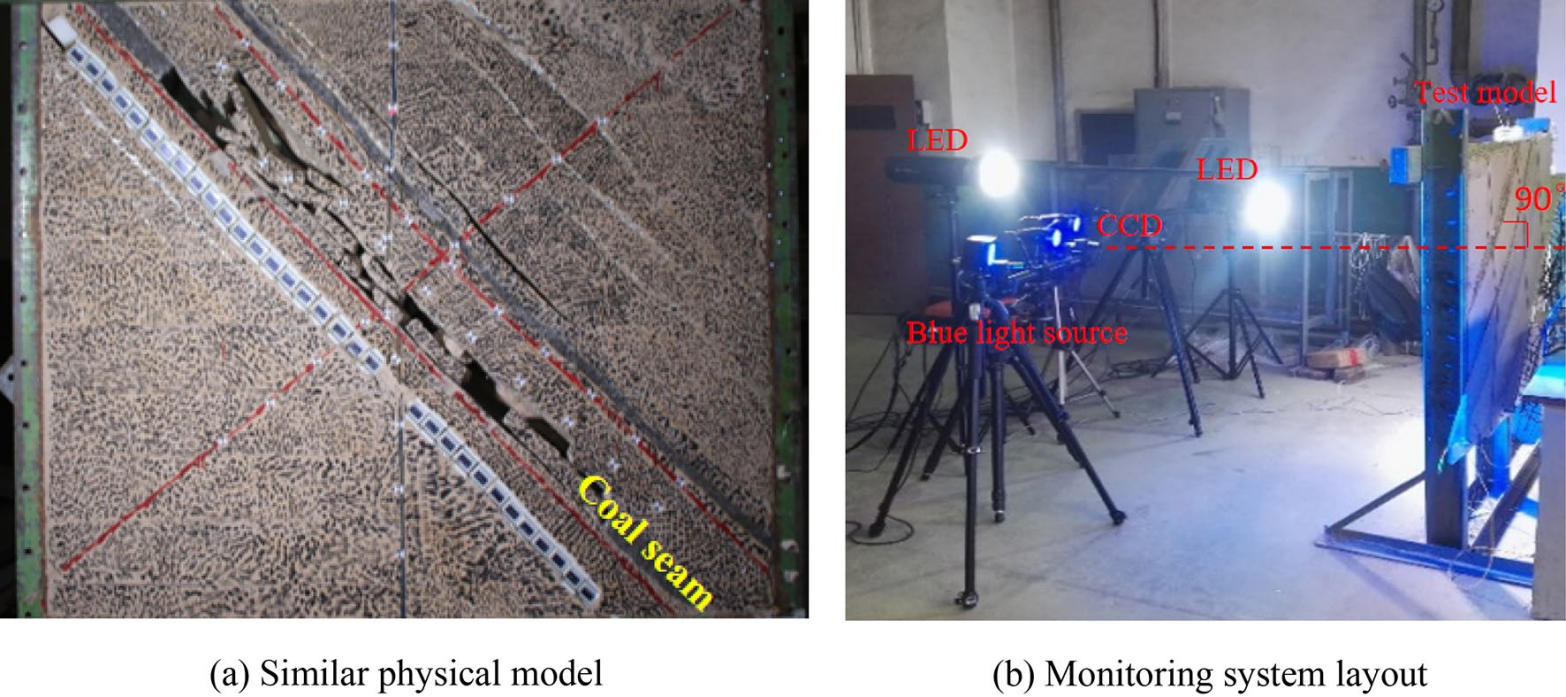
Two-dimensional similar physical model and test.

Similar materials are usually prepared from several materials. According to the above requirements and previous research experience, it is determined that river sand, fly ash, and mica (also used as layered materials) are used as aggregates, gypsum and white powder are used as cementitious materials, and water is used as solvent materials for each rock (coal) layer in this test. After selecting similar materials, according to the determined similarity constant and the physical and mechanical properties of each rock stratum, the ratio is adjusted through the material ratio experiment to meet the requirements of mechanical similarity (Table 1). Similar materials shall be laid layer by layer according to equal proportions. The similarity model after mining is shown in Fig. 10a. The excavation step of the working face is 30 mm, and the mining height is 40 mm. The mining of the simulated working face has been excavated 30 times, with a total advance of 900 mm.

**Table 1:** Physical and mechanical properties of the strata.

Layer number	Lithology	Thickness/m	Thickness of the model/cm	Ratio (sand: gypsum: CaCO3: coal)	Sand/kg	Gypsum/kg	CaCO_3_/kg
1	Loess	42.00	28.0	1019	238.25	2.38	21.44
2	Sandy mudstone	14.76	9.8	919	82.89	0.92	8.29
3	Siltstone	21.55	14.4	837	119.53	4.48	10.46
4	Medium sand	28.75	19.2	837	159.47	5.98	13.95
5	Siltstone	6.70	4.5	937	37.63	1.25	2.93
6	Medium sand	9.96	6.6	828	55.24	1.38	5.52
7	1^–2^ Coal seam	1.89	1.3		5.13	0.19	1.28
8	Fine sandstone	2.85	1.9	937	16.01	0.53	1.24
9	Fine sandstone	6.55	4.4	837	36.33	1.36	3.18
10	Siltstone	3.80	2.5	828	21.08	0.53	2.11
11	Fine sandstone	5.90	3.9	837	32.73	1.23	2.86
12	Siltstone	1.00	0.7	937	5.62	0.19	0.44
13	Fine sandstone	11.00	7.3	828	61.01	1.53	6.10
14	Fine sandstone	2.16	1.4	937	12.13	0.27	1.08
15	2^-2^Coal seam	4.60	3.1		12.48	0.47	3.12
16	Siltstone	3.54	2.4	937	19.88	0.66	1.55
17	Fine sandstone	8.70	5.8	837	48.26	1.81	4.22

### Monitoring system layout

Figure 10b shows the DIC test system, including an industrial camera (2560 × 1920 pixel), LED light source, computer, and 2D-DIC analysis software. Because the test object is a two-dimensional plane similar material model and does not pay attention to the out-of-plane displacement of model deformation, the selection of 2D-DIC test system in this paper can reduce the test cost and improve the test efficiency.

Adjust the camera position so that the camera shooting direction is perpendicular to the model surface. The camera is controlled by an infrared remote controller, which can avoid camera vibration caused by manual operation. Two groups of LED light sources provide uniform and bright lighting for the model surface. The proportional relationship between the pixel and the actual length in the calibration image and its magnification is 2.882 pixel / mm. Using the parameter setting of subset size length of 40 pixels and subset size spacing of 10 pixels for DIC processing, the displacement of all measurement points in the analysis area on the image is obtained. The calculation error caused by model jitter can be eliminated by using rigid body motion compensation in 2D-DIC software. Taking the photos of the model surface before model excavation as the reference image, the images of the model surface under different excavation states are analyzed by 2d-DIC software to obtain the deformation information of the model surface under other excavation states.

To quantitatively evaluate the precision of the DIC measurement system, one of the two images in the unloaded stage is selected as the reference image for DIC processing. The displacement of all measurement points in the analysis area on the picture is obtained, and the standard deviation of the displacement is obtained. Since the actual removal is zero, the standard deviation of the measured displacement can be defined as:

5 $$ \sigma { = }\sqrt {\frac{1}{N - 1}\sum\nolimits_{i = 1}^{N} {d_{i}^{2} } } $$

where *d*_*i*_ is the displacement of each measuring point in the image, and *N* is the number of measuring points, with 35,931 measuring points. The calculation results reflect the size of random error in DIC measurement.

The calculated displacement standard deviation is 0.007 mm, which shows the rationality of the layout and parameter setting of the DIC measurement system. The DIC measurement system can provide high spatial resolution and meet the accuracy requirements of similar material model monitoring. Compared with the total station based on point measurement, DIC measurement technology has the advantages of being short time-consuming and high efficiency^37,38^. It can more truly and comprehensively reflect the movement and deformation of surface and rock stratum in the process of mining.

## Test results and analysis

### Development characteristics of complex multi-fracture

**Figure 11:**
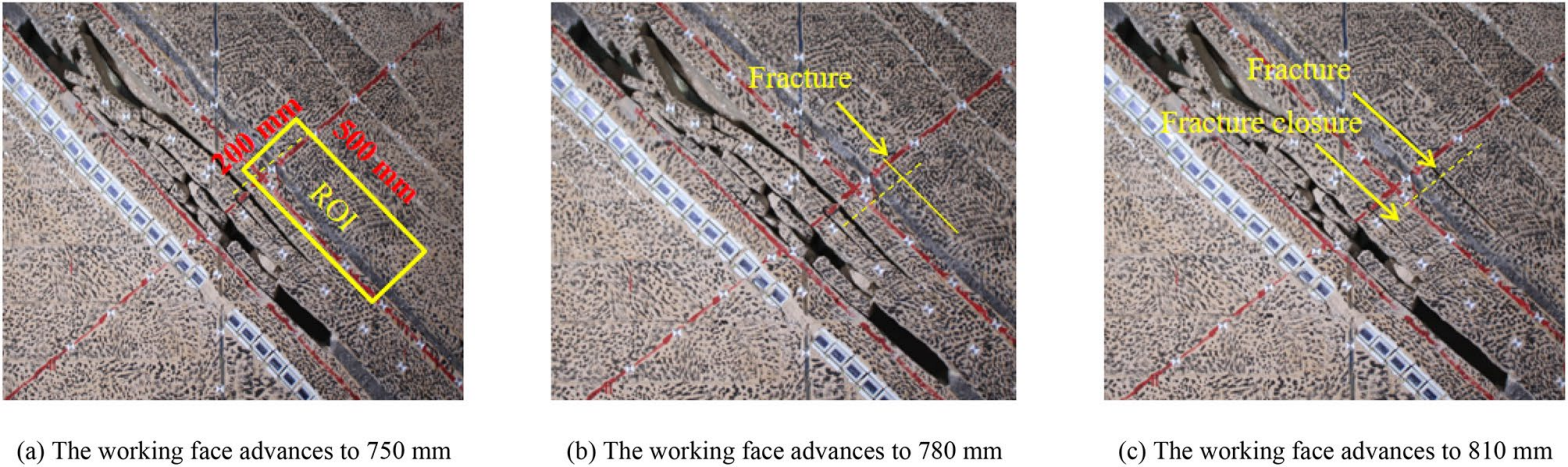
Development characteristics of complex multi-fracture.

As shown in Fig. 11 shows the development characteristics of complex and multi-fractures. In the process of actual rock fracture development, it is often accompanied by the opening and closing of other fractures. When the working face advances to 750 mm, multiple fractures appear in the upper part of the basic roof rock, of which the most developed fracture length is 468.98 mm, and the maximum the crack opening displacement is 33.69 mm. Cracks are generated under the rock layer, and the collapsed rock layer is hinged with the previously broken rock beam to form a masonry beam structure. When the working face advances to 780 mm, the collapsed gangue in the goaf and the unbroken rock layer above the working face squeeze each other, which prevents the normal collapse of the direct roof rock layer to a certain extent. The fissure continues to develop, but the change is minimal. The development length of the crack is 471.32 mm, and the maximum crack opening is 34.25 mm. When the working face advances to 810 mm, the fracture formed by the last advance is closed, and new fractures are developed in the roof above the coal seam. The crack development length is 290.8 mm, and the maximum crack opening is 7.43 mm. Use a micrometer to measure the crack opening at the yellow dotted line in Fig. 11. When the working face is pushed to 750 ~ 810 mm, the measured crack opening is 0.61 mm, 0.83 mm, and 6.51 mm, respectively. As shown in Fig. 11a, the rectangular area 200 × 500 mm above the working face is selected as the main analysis area in the region of interest (ROI) for the following DIC calculation. As shown in Fig. 11b, with the continuous advancement of the working face, the roof above the crack to be measured changes little, while the development length and opening of the crack to be measured gradually expand with the continuous advancement of the working face. When the advancing distance of the working face reaches 810 mm, the crack below the crack is closed.

### Measurement results of traditional method

**Figure 12:**
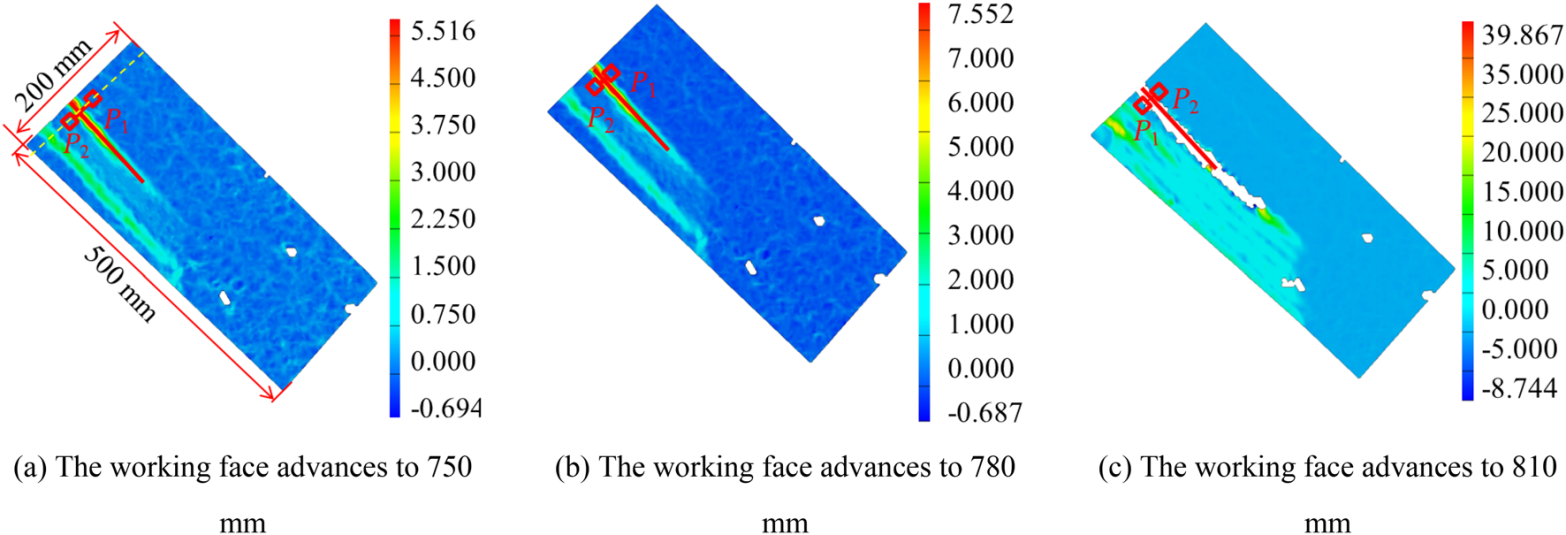
Layout of traditional method measuring points.

Select above the working face 200 × 500 mm Perform DIC calculation in analysis area, as shown in Fig. 12a. Figure 12 is the strain field program obtained by DIC when the working face is pushed to 750 ~ 810 mm. The virtual extensometer method is used to measure the crack opening at the yellow dotted line. Two measuring points P1 and P2, are symmetrically arranged on both sides of the strain localization zone, taking a = 3 mm, and taking each measuring point as the center, taking five groups of pixel points as the calculation area to calculate the crack opening.

When the working face is pushed to 750 ~ 810 mm, the corresponding crack opening is 0.58 mm, 0.72 mm, and 5.13 mm, respectively. When the work is advanced to 750 mm, the relative error between the the crack opening displacement obtained by the virtual extensometer method and the measured the crack opening displacement is 4.91%. When the work is advanced to 780 mm, the relative error between the the crack opening displacement obtained by the virtual extensometer method and the measured the crack opening displacement is 13.25%. When the work is advanced to 810 mm, the relative error between the the crack opening displacement obtained by the virtual extensometer method and the measured the crack opening displacement is 21.20%. It can be found that with the increase of the advancing distance of the working face, the relative error between the the crack opening displacement obtained by the virtual extensometer method and the measured the crack opening displacement gradually increases because when the working advances to 750 mm, the calculation area of the measuring point P1 covers the non-uniform deformation area in part of the strain localization zone, The calculation region where the measuring point P2 is located is in the uniform deformation region outside the strain localization band. When the work is advanced to 780 mm, the calculation area of measuring points P1 and P2 covers the non-uniform deformation area in some strain localization bands. When the work is advanced to 810 mm, the calculation area of measuring points P1 and P2 partially covers the fracture area.

In complex fracture propagation, the traditional method is difficult to accurately measure the the crack opening displacement^39–42^. The test results verify the influence of the relative distance between the measuring point layout and the crack on the the crack opening displacement measured by DIC.

### Measurement results of improved method

**Figure 13:**
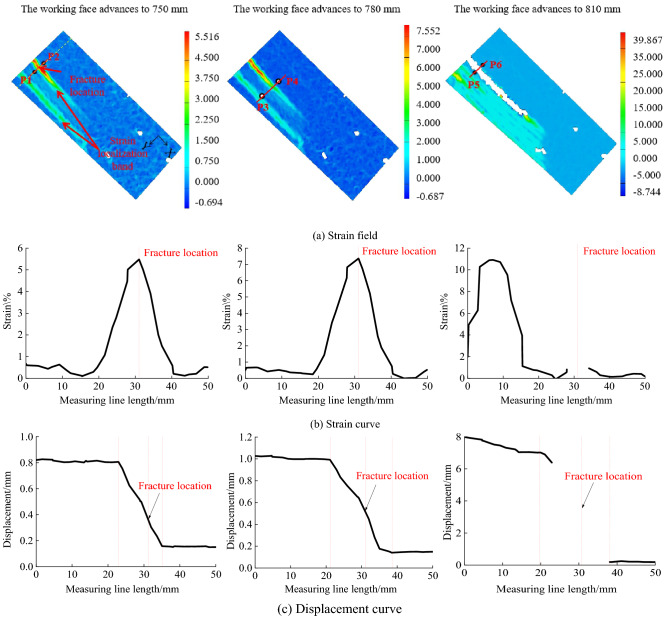
Layout of improved method measuring points.

As shown in Fig. 13, the improved method is used to measure the crack opening at the yellow dotted line. Firstly, the measuring line perpendicular to the strain localization zone is arranged at the yellow dotted line. Then the strain change curve and displacement change curve of each point along the measuring line is measured by DIC. The abscissa is the distance of the measuring point, with a maximum of 50 mm.

Figure 13 is the cloud diagram of the DIC strain field obtained when the working face is pushed to 750 ~ 810 mm and the strain change curve and displacement change curve measured by DIC along the measuring line. In Fig. 13b, when the working face is pushed to 750 mm, the strain curve of the measuring line in the displacement change curve is distributed in a single peak shape at the position of 31 mm, which corresponds to the crack position in the strain field cloud diagram when the working face is pushed to 750 mm in Fig. 13a. Figure 13 (c) is the displacement change curve of each point along the measuring line measured by DIC, and the displacement curve is distributed in steps as a whole.

When the working face is pushed to 750 mm, the measuring line is in the range of 0 ~ 23 mm and 35 ~ 50 mm, and the overall change of displacement is small. The measuring line is located in the uniform deformation area outside the strain localization zone within this range. The displacement of the measuring line varies sharply in the range of 23 ~ 35 mm, and the measuring line is located in the non-uniform deformation area in the strain localization zone. The measuring points shall be arranged in the uniform deformation area outside the strain localization zone. The distance between the measuring point P1 and the crack is 9 mm, and the distance between the measuring point P2 and the crack is 6 mm.

When the working face is pushed to 780 mm, the measuring line is in the range of 0 ~ 21 mm and 38 ~ 50 mm, and the overall change of displacement is small. The measuring line is located in the uniform deformation area outside the strain localization zone within this range. The displacement of the measuring line varies violently within the range of 21 ~ 38 mm. The measuring line is located in the non-uniform deformation area in the strain localization zone within this range. The measuring points should be arranged in the uniform deformation area outside the strain localization zone. The distance between measuring point P3 and the crack is 12 mm, and the distance between measuring point P4 and the crack is 8 mm.

When the working face advances to 810 mm, multiple strain localization zones appear in the strain field program due to the movement and failure of overburden. The measuring line is in the range of 14 ~ 19 mm and 38 ~ 50 mm, and the overall change of displacement is small. The measuring line is located in the uniform deformation area outside the strain localization zone within this range. When the measuring line is 0 ~ 14 mm, the displacement changes sharply. The measuring line is located in the non-uniform deformation area in the strain localization zone within this range. When the measuring line is 19 ~ 38 mm, the displacement data is missing. Within this range, the measuring line is located in the fracture area. The measuring points shall be arranged in the uniform deformation area outside the strain localization zone. The distance between measuring point P5 and the crack is 13 mm, and the distance between measuring point P6 and the crack is 10 mm.

Adjust the direction of the coordinate system so that the Y-axis of the coordinate system is perpendicular to the crack direction. It is calculated that when the working face is pushed to 750 ~ 810 mm, the corresponding crack opening is 0.63 mm, 0.86 mm, and 6.73 mm, respectively. The maximum relative error between the the crack opening displacement obtained by the improved method and the measured the crack opening displacement is 3.61%. In the improved method, the layout position of the measuring points is adjusted with the change of the width of the strain localization zone to ensure that the measuring points are always located in the uniform deformation area outside the strain localization zone. Therefore, the measured results are consistent with the measured crack opening.

Comparing Fig. 11 and Fig. 13, it can be found that the final expansion path of strain localization zone and fracture is consistent, and the width of strain localization zone also changes with the development of fracture, which is consistent with the previous research results. Compared with the traditional method, the improved method not only considers the influence of strain localization zone and fracture on the displacement error of measuring points but also considers the temporal and spatial characteristics of fracture development in the process of complex fracture propagation, which can realize the reasonable layout of measuring points under multiple cracks and multiple strain localization zones. In complex multi-fracture propagation, the improved method has better accuracy than the traditional method.

## Conclusion

In this paper, through theoretical analysis, numerical simulation test of strain localization band, indoor single crack test and two-dimensional similar model test of complex multi cracks, and analyzing the Spatio-temporal evolution process of complex multi cracks, an improved virtual extensometer measurement method is proposed, and the measurement results are compared with those of traditional methods. The results show that:

1. The measurement accuracy of the virtual extensometer measuring point in the strain localization zone increases with the subset size and spacing decrease. The measurement accuracy of virtual extensometer points outside the strain localization region increases with the subset size and spacing increase.
2. In the case of complex multi fractures, to accurately obtain the crack opening displacement, the relative distance between the virtual extensometer measuring point and the crack should be greater than half of the sum of the width of the crack strain localization zone and the subset size. With the development of the crack, the relative distance between the virtual extensometer measuring point and the crack should increase with the increase of the width of the crack strain localization zone.
3. Compared with the traditional method, the improved method not only considers the influence of strain localization zone and fracture on the displacement error of measuring points but also considers the temporal and spatial characteristics of fracture development in the process of complex fracture propagation, which can realize the reasonable layout of measuring points under multiple cracks and multiple strain localization zones.
4. The error of the crack opening displacement measured by the traditional method increases with the fracture development, and the maximum is 21.20%. The maximum relative error between the crack opening measured by the improved method and the measured crack opening is 3.61%, which verifies the reliability of the improved method in the process of complex fracture propagation.
